# Mammalian Target of Rapamycin (mTOR) and the Proteasome Attenuates IL-1β Expression in Primary Mouse Cardiac Fibroblasts

**DOI:** 10.3389/fimmu.2019.01285

**Published:** 2019-06-06

**Authors:** May-Kristin Torp, Kuan Yang, Trine Ranheim, Knut Husø Lauritzen, Katrine Alfsnes, Leif E. Vinge, Pål Aukrust, Kåre-Olav Stensløkken, Arne Yndestad, Øystein Sandanger

**Affiliations:** ^1^Division of Physiology, Department of Molecular Medicine, Faculty of Medicine, Institute of Basic Medical Sciences, University of Oslo, Oslo, Norway; ^2^Centre for Heart Failure Research, University of Oslo, Oslo, Norway; ^3^Research Institute of Internal Medicine, Oslo University Hospital Rikshospitalet, Oslo, Norway; ^4^Department of Internal Medicine, Diakonhjemmet Hospital, Oslo, Norway; ^5^Section of Clinical Immunology and Infectious Diseases, Oslo University Hospital Rikshospitalet, Oslo, Norway; ^6^Faculty of Medicine, Institute of Clinical Medicine, University of Oslo, Oslo, Norway; ^7^Section of Dermatology, Oslo University Hospital Rikshospitalet, Oslo, Norway

**Keywords:** IL-1, NLRP3, inflammasome, mTOR, proteasome, cardiac, fibroblasts, chloroquine

## Abstract

**Background:** IL-1β is a highly potent pro-inflammatory cytokine and its secretion is tightly regulated. Inactive pro-IL-1β is transcribed in response to innate immune receptors activating NFκB. If tissue damage occurs, danger signals released from necrotic cells, such as ATP, can activate NLRP3-inflammasomes (multiprotein complexes consisting of NLRP3, ASC, and active caspase-1) which cleaves and activates pro-IL-1β. NLRP3 activation also depends on NEK7 and mitochondrial ROS-production. Thus, IL-1β secretion may be regulated at the level of each involved component. We have previously shown that NLRP3-dependent IL-1β release can be induced in cardiac fibroblasts by pro-inflammatory stimuli. However, anti-inflammatory mechanisms targeting IL-1β release in cardiac cells have not been investigated. mTOR is a key regulator of protein metabolism, including autophagy and proteasome activity. In this study we explored whether autophagy or proteasomal degradation are regulators of NLRP3 inflammasome activation and IL-1β release from cardiac fibroblasts.

**Methods and Results:** Serum starvation selectively reduced LPS/ATP-induced IL-1β secretion from cardiac fibroblasts. However, no other inflammasome components, nor mitochondrial mass, were affected. The mTOR inhibitor rapamycin restored pro-IL-1β protein levels as well as LPS/ATP-induced IL-1β release from serum starved cells. However, neither serum starvation nor rapamycin induced autophagy in cardiac fibroblasts. Conversely, chloroquine and bafilomycin A (inhibitors of autophagy) and betulinic acid (a proteasome activator) effectively reduced LPS-induced pro-IL-1β protein levels. Key findings were reinvestigated in human monocyte-derived macrophages.

**Conclusion:** In cardiac fibroblasts, mTOR inhibition selectively favors pro-IL-1β synthesis while proteasomal degradation and not autophagy is the major catabolic anti-inflammatory mechanism for degradation of this cytokine.

## Introduction

Interleukin-1β (IL-1β) is a highly potent inflammatory cytokine critical for host defense against infections, but could also promote harmful effects on the host if the response is overwhelming or too long. Its secretion is therefore tightly regulated through inflammasome-mediated post-translational activation. During sterile inflammatory responses, this most commonly involves the NLRP3 (i.e., Nod-like receptor with a PYD-domain 3) inflammasome. Inactive pro-IL-1β is transcribed and translated in response to innate immune receptors and cytokine receptors activating the transcription factor Nuclear Factor κB (NFκB) (signal 1). For activating pro-IL-1β, a second signal is needed consisting of a wide range of chemically different danger signals such as extracellular ATP and urate crystals that activate NLRP3 (signal 2). Signal pathways activating NLRP3 all seem to converge at a state of reduced cytosolic potassium concentration and mitochondrial reactive oxygen species (ROS) formation (1). Recently, NIMA-related kinase 7 (NEK7) was reported to be the long sought endogenous ligand of NLRP3 by three independent research groups (2–4). NLRP3 activation leads to formation of the multiprotein complexes termed inflammasomes, consisting of NLRP3, ASC and caspase-1. Pro-IL-1β is the main substrate of caspase-1, and IL-1β secretion is the gold standard readout of NLRP3 inflammasome activity.

IL-1β is highly expressed in cardiac tissue after acute myocardial infarction (MI) (5), even measurable in patient's plasma the first 5 h post-MI (6). Experimental mouse models have shown that IL-1β is of major importance for recruiting leukocytes, especially neutrophils and monocytes, to the infarcted area post-MI (5, 7). Moreover, blockade of IL-1 signaling has been reported to attenuate post-MI heart failure in both mice and men (7, 8). We have previously shown that functional NLRP3 inflammasomes can be induced in cardiac fibroblasts by inflammatory stimuli. Furthermore, NLRP3 deficient hearts were protected against ischemia-reperfusion mediated tissue damage in an *ex vivo* Langendorff model (9). Thus, IL-1β and the NLRP3 inflammasome are thought to contribute to post-MI tissue damage and adverse remodeling. Catabolic removal of inflammasome proteins, as well as mitochondria and the substrate pro-IL-1β may serve as anti-inflammatory mechanism. Indeed, removal of pro-IL-1β and mitochondria by autophagy has been reported to attenuate IL-1β release from macrophages *in vitro* (10, 11). The key regulator of anabolism vs. catabolism, including autophagy and proteasomal degradation, is mammalian target of rapamycin (mTOR) (12–15). However, anti-inflammatory catabolism targeting the NLRP3-dependent IL-1β release has not been explored in cardiac cells.

In this study we explored the role of NLRP3 inflammasome protein catabolism in primary cardiac fibroblasts as a possible anti-inflammatory mechanism. We found that pro-IL-1β is the main and only target of starvation-induced catabolism. Surprisingly, mTOR inhibition with rapamycin, a known inducer of autophagy, did not affect autophagy in cardiac fibroblasts, and favored pro-IL-1β synthesis. However, the autophagy inhibitor chloroquine effectively degraded pro-IL-1β in both cardiac fibroblasts and human macrophages, potentially also involving enhanced proteasomal activity.

## Materials and Methods

### Reagents

Ultra-pure lipopolysaccharide (LPS, 0111:B4) from *Escherichia coli*, rapamycin, chloroquine, MG-132, and bafilomycin A1 were purchased from Invivogen (Carlsbad, CA). ATP, betulinic acid, and staurosporine were purchased from Sigma-Aldrich (St. Louis, MO). MitoTracker Deep Red FM was purchased from Thermo Fisher Scientific (Massachusetts, United States). Antibodies are listed in Table 1.

**Table 1:** Antibodies used.

**Antibody**	**Source/company**	**Application**
Anti-NEK7 (Ab133514)	Abcam	Western blot
Anti-NEK7 (bs7758R-A488)	Bioss	Confocal microscopy
Anti-NLRP3 (D2P5E)	Cell signaling	Western blot
Anti-ASC (D2W8U)	Cell signaling	Western blot
Anti-ASC (bs6741R-A647)	Bioss	Confocal microscopy
Anti-procaspase-1 (Ab1872)	Abcam	Western blot
Anti-pro-IL-1β (AF-401-NA)	R&D	Western blot
Anti-SDHA (complex II) (Ab14715)	Abcam	Western blot
Anti mTOR (7C10)	Cell signaling	Western blot
Anti phospho-mTOR (Ser2448) (D9C2)	Cell signaling	Western blot
Anti p70 S6 kinase (#9202)	Cell signaling	Western blot
Anti phospho-p70 S6 kinase (Thr389) (#9205)	Cell signaling	Western blot
Anti ubiquitin (P4D1)	Cell signaling	Western blot
Anti LC3B (D11) (#3868)	Cell Signaling	Immune fluorescence microscopy
Anti-rabbit IgG HRP (7074S)	Cell signaling	Western blot
Anti-mouse IgG HRP (7076S)	Cell signaling	Western blot
Anti-goat IgG HRP (sc-2020)	Santa cruz	Western blot
Anti-cleaved Caspase-8 (Asp387)(#9429)	Cell signaling	Western blot

### Isolation and Culture of Mouse Cardiac Fibroblasts

Mice were anesthetized by intraperitoneal (i.p.) injection of sodium pentobarbital (50 mg/kg) and heparinized (500 IU, Leo Pharma A/S, Denmark) before they were euthanized by cervical dislocation.

Hearts were retrogradely perfused with perfusion buffer containing (in mM): NaCl: 120.4; KCl: 14.7; KH_2_PO_4_: 0.6; Na_2_PO_4_: 0.6; MgSO_4_: 1.2; Na-HEPES liquid: 10.0; Glucose: 5.5; NaHCO_3_: 4.6; Taurine: 30.0; BDM (2,3-Butanedione monoxime): 10. Heart perfusion was initially with perfusion buffer alone, then with addition of 1.3 mg/mL Collagenase type 2 (#4177, batch 45D15719, activity 355 U/mg, Worthington Biochemical, Lakewood, NJ, USA). After collagenase inhibition with HyClone® Bovine Calf Serum (FBS, #SH30073.03, GE Healthcare Life Sciences, Marlborough, MA, USA) diluted in perfusion buffer, cells were separated by gently pipetting and centrifugation at 20 × g for 2 min to separate cardiomyocytes from non-cardiomyocytes. The non-cardiomyocyte suspension was further centrifuged twice at 800 × g for 3 min, before culturing in uncoated T75 flasks with fibroblast medium (DMEM, low glucose, pyruvate (#31885, Gibco, Thermo Fisher Scientific, Massachusetts, United States) supplemented with 10% FBS (Biowest #S1181.B, Nuaillé, France), and 100 U/mL penicillin-streptomycin, and incubated at 37°C and 5 % CO_2_. Medium was changed every 3–4 days and the cardiac fibroblasts were grown to confluency before collected and utilized in experiments at first passage. The day before the experiments, the cells were seeded into Nunclon Delta Surface wells (Thermo Scientific, Denmark) (for western blot analysis: 6-well plates, 120,000 cells/well in 2 mL medium; for mRNA quantification and ELISA: 12-well plates, 47,000 cells/well in 1 mL medium. Unless other is specified in the figure legends, the following concentrations of reagents were used in the experiments: LPS: 10 ng/mL; rapamycin 500 nM; chloroquine: 20 μM; ATP: 3 mM. Time is specified in figure legends.

### Isolation of Human Monocytes and Macrophage Differentiation

Monocytes were isolated from whole blood from healthy donors by Lymphoprep (Axis-Shield, Oslo, Norway) and plastic adherence. Monocytes were cultured in RPMI 1640 (PAA Laboratories, Pasching, Austria) containing 10% FCS, 5 U penicillin/ml and 50 μg/ml streptomycin (P/S) (Sigma-Aldrich). For macrophage differentiation, monocytes were incubated with 20 ng/mL human M-CSF (R&D Systems Minneapolis, MN) for 7 days in RPMI 1,640 with 10% FCS. Medium was replaced day 3 and 6.

### Culture of HL-1 Cells

HL-1 cells were purchased from Sigma-Aldrich and cultured in Claycomb medium (Sigma-Aldrich) with 10% FCS.

### Quantification of mRNA Levels

Total RNA was isolated from cultured cells with the RNeasy Mini Kit (Qiagen, Hilden, Germany) and quantified with ND-1000 Spectrophotometer (NanoDrop, Thermo Fisher Scientific, Rockford, IL). cDNA was made using qScript^TM^ cDNA SuperMix (Quanta Biosciences, Beverly, MA). Quantification of gene expression was performed using the Stratagene MX3005P (Agilent Technologies, Cedar Creek, TX), PerfeCTa SYBRGreen FastMix Low Rox (Quanta Biosciences), and sequence-specific PCR primers designed using the Primer Express software, version 3.0 (Applied Biosystems, Foster City, CA). Gene expression of the glyceraldehyde 3-phosphate dehydrogenase (GAPDH) was used as reference for relative quantifications. Target gene expressions in controls were defined as one, and normalization performed accordingly. Primer sequences are listed in Table 2.

**Table 2:** Primer sequences.

**Target**	**Species**	**Sequence (5′ 3′)**	**Acc.nr**
IL-1β	Mouse	(+)-GCCACCTTTTGACAGTGATGAG	NM_008361
		(–)-GTTTGGAAGCAGCCCTTCATC	
IL-1β	Human	(+)-CCCTAAACAGATGAAGTGCTCCTT	NM_000576
		(–)-GGTGGTCGGAGATTCGTAGCT	
TNF	Mouse	(+)-AGACCCTCACACTCAGATCATCTTC	NM_013693
		(–)-CCACTTGGTGGTTTGCTACGA	
TNF	Human	(+)-CCAGGCAGTCAGATCATCTTCTC	NM_000594
		(–)-GGAGCTGCCCCTCAGCTT	
GAPDH	Mouse/rat/ human	(+)-CCAAGGTCATCCATGACAACTT (–)-AGGGGCCATCCACAGTCTT	NM_008084

*IL-1β, interleukin 1β; TNF, Tumor necrosis factor; GAPDH, Glyceraldehyde 3-phosphate dehydrogenase*.

### Quantification of Cytokines With Multiplex Analysis and ELISA

Mouse IL-1β, and tumor necrosis factor (TNF), macrophage inflammatory protein (MIP)-2 and interleukin (IL)-6 were quantified with duoset ELISA (R&D Systems, Minneapolis, MN).

### Immune Fluorescence Microscopy and LC3B Puncta Quantification

To evaluate the LC3B dots in cardiac fibroblasts, 15,000 cells were cultured in Lab-Tek II eight well glass chamber slides (Thermo Fisher Scientific Inc.,). After experimental interventions, the cells were fixed with 2% paraformaldehyde in PBS on ice for 15 min before permabilization and blocking in 0.2% saponin/5% BSA/PBS in room temperature for 20 min. Cells were then incubated over night at +4°C in 5 μg/mL LC3B antibody diluted in 0.2% saponin/5% BSA/PBS. 5 μg/mL anti-rabbit Alexa488 secondary antibody (Thermo Fisher Scientific Inc.,) and 3.2 μM Hoechst 33258 (Thermo Fisher Scientific Inc.,) were added and incubated for 1 h at room temperature. Gelatine-glycerol mounting medium, pre-heated to 55°C was added to the slide, which were sealed with a cover glass. The slides were analyzed with Zeiss high-throughput microscope (Carl Zeiss AG, Oberkochen, Germany) at 20 × magnification. Forty-nine images were automatically captured per well and number of nuclei and number of LC3B dots were analyzed with Cell profiler™ cell image analysis software (16).

### Confocal Imaging

For confocal imaging, 12,000 cells were seeded in 100 μL medium on the glass area of 35 mm glass-bottom gamma-irradiated dishes (MatTek Corporation, Ashland, MA), after 1 h 1 mL medium was added. Cell confluences were 80–90% at the time the experiments started. For intracellular imaging, cells were fixed with 3% paraformaldehyde/PBS on ice for 15 min, washed once with 1% FCS/PBS, permeabilized with 0.1% saponin/5% bovine serum albumin (BSA)/PBS at room temperature for 20 min, then incubated with the appropriate antibodies (5 μg/ml in 0.1% saponin/1% BSA/PBS) for 45 min at room temperature. The cells were then washed three times with 0.1% saponin/1% BSA/PBS before incubation with 2 ml PBS. Images were captured with a Zeiss Elyra-S microscope with a 63 x objective.

### Flow Cytometry

For flow cytometry analysis of MitoTracker staining, 120,000 cardiac fibroblasts were seeded per well in 6 well plates and incubated with 10% FCS/DMEM for 24 h. The cells were then incubated with 10 ng/mL LPS with or without 10% FCS in low glucose DMEM (Gibco, Thermo Fisher Scientific) for 20 h, then incubated with 500 nM MitoTracker Deep Red FM (Thermo Fisher Scientific) in DMEM without serum for 45 min. The cells were washed with PBS, detached with trypsin/EDTA and trypsin subsequently inactivated with 10% FCS in DMEM. The cells were washed once with ice cold PBS and kept on ice until analysis with a MACSQuant Analyzer 10 (Miltenyi Biotec, Bergisch Gladbach, Germany).

### Western Blot and Protein Quantification

Cells were lysed in M-PER™ Mammalian Protein Extraction Reagent (78501; Thermo Scientific) supplemented with protease inhibitors (Complete Protease Inhibitor Cocktail, Roche Applied Science, Mannheim Germany). Protein homogenates were separated under denaturing conditions on Any-KD or 10% SDS-polyacrylamide gels (Mini-PROTEAN Precast gels; Bio-Rad, Hercules, CA) and electro-blotted on to PVDF membranes. For pro-IL-1β detection, the membranes were blocked in Superblock T20 (Thermo Fisher Scientific) and incubated with 0.1 μg/ml goat anti-mouse IL-1β antibody (AF-401-NA; R&D Systems) diluted in 20% Superblock T20/TBST, and subsequently a horseradish peroxidase-conjugated donkey anti-goat antibody (Santa Cruz Biotechnology, Santa Cruz, CA). Other membranes were blocked with 5% dry milk/TBST and proteins were incubated with 5% dry milk/TBST or 5% BSA/TBST according to manufacturer's protocol. Protein expression was detected by chemiluminescence (SuperSignal West Pico; Thermo Fisher Scientific). Protein quantifications were done with the ImageJ software.

### Ethics

The part of the study that included human monocytes was approved by the local ethical committee (Regional ethics committee of Helse Sør-Øst; Permit number S-05172) and conducted according to the ethical guidelines outlined in the Declaration of Helsinki for use of human tissue and subjects. Animal experiments (isolation of primary mouse cardiac fibroblasts) were approved by the Norwegian Animal Research Authority 80 project license no FOTS id 7,333 and 13,643. The isolations of primary animal cells were performed in accordance with the European Directive 2010/63/EU and The Guide for the Care and Use of Laboratory Animals, 8th edition (NRC 2011, National Academic Press). In line with the ethics of the Norwegian Animal Research Authority, the number of biological repeats were kept as small as possible in order to minimize the number of sacrificed animals.

### Statistics

For comparisons of two groups, the paired student *t*-test were performed. Probabilities are two-sided and considered to be significant when *p* < 0.05.

## Results

### IL-1β Release From Cardiac Fibroblasts Depends on Mitochondrial ROS and Is Attenuated by Serum Starvation

We hypothesized that NLRP3-dependent IL-1β secretion can be negatively regulated by autophagic degradation of the inflammasome proteins in cardiac fibroblasts. The classical NLRP3 inflammasome components are NLRP3, ASC and caspase-1. Furthermore, NEK7 was recently reported to be an endogenous NLRP3 agonist in mouse bone marrow derived macrophages by three independent research groups (2–4). In accordance with this, confocal microscopy showed NEK7 co-localizing with ASC in cardiac fibroblasts primed with LPS and activated with ATP (Supplementary Figure 1A). Thus, we also considered NEK7 as a potential target for NLRP3 inflammasome regulation. Finally, several studies have supported that mitochondrial ROS is essential for NLRP3 activation in macrophages (17–19). Indeed, the mitochondrial targeted ROS scavenger MitoTempo completely inhibited IL-1β release from cardiac fibroblasts while TNF secretion was not affected (Supplementary Figure 1B), suggesting mitophagy as a possible regulatory mechanism for IL-1β release.

**Figure 1:**
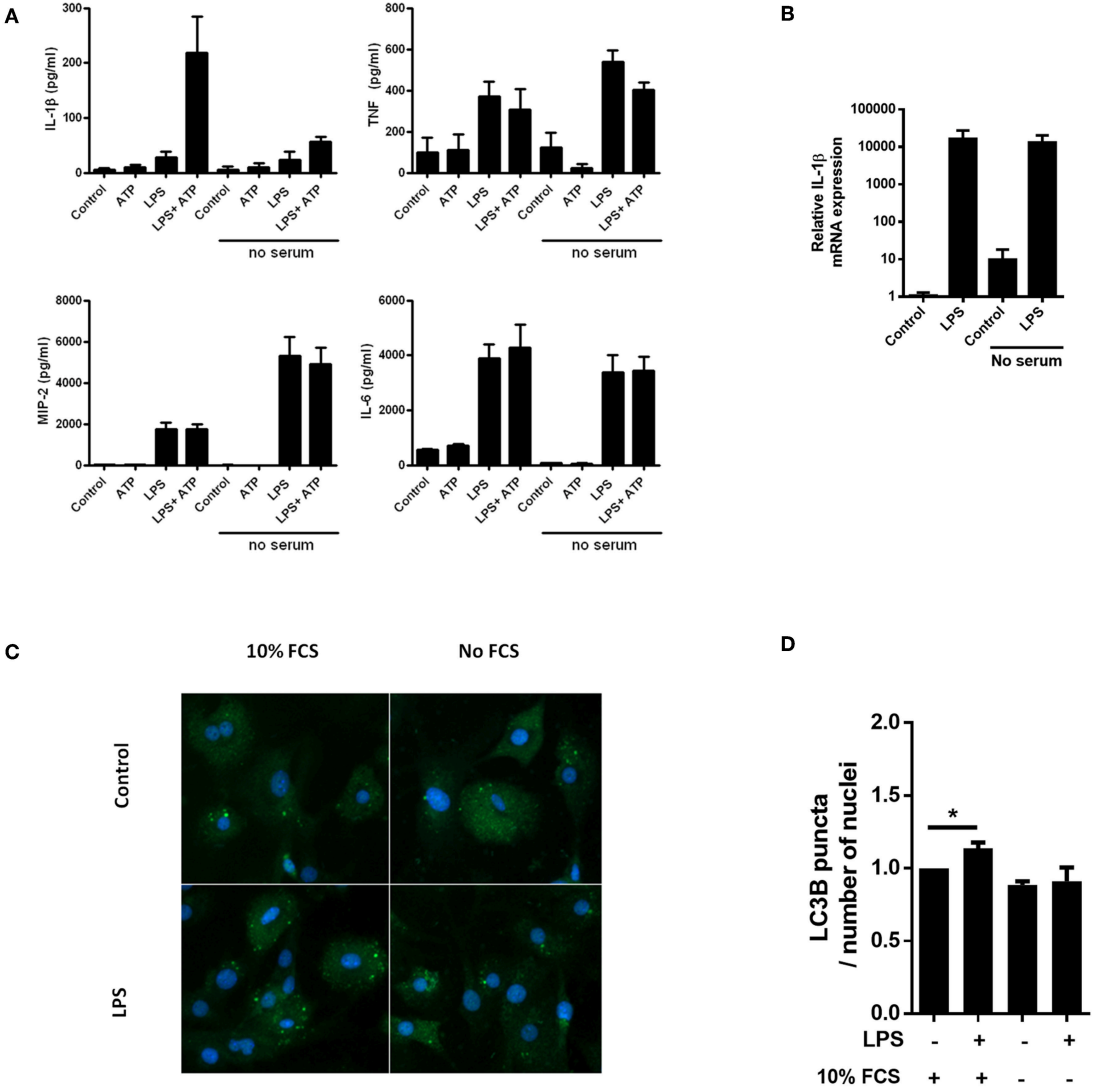
Lack of serum selectively attenuates IL-1β release from cardiac fibroblasts with no induction of autophagy. **(A)** Cardiac fibroblasts were incubated with 10 ng/mL LPS for 20 h with or without 10% heat inactivated fetal calf serum, then incubated with or without 3 mM ATP for 60 min. Cytokines were quantified in conditioned media (*n* = 5). **(B)** In the same experiment as **(A)**, pro-IL-1β mRNA were quantified with PCR. Data are normalized to control = 1. **(C)** Cardiac fibroblasts primed with LPS (10 ng/mL) were incubated with 10% FCS (control) or no serum for 4 h. Cells were labeled with anti-LC3B and Hoechst and whole slides scanned with an automated immunofluorescence microscope. **(D)** LC3B puncta in all cells were automatically counted and the ratio to the number of Hoechst-labeled kernels calculated. Paired data were normalized to control = 1 (*n* = 5). All columns are mean with SEM. ^*^p < 0.05 (paired student *t*-test).

Serum starvation can be a powerful inducer of autophagy (20). In accordance with our hypothesis, we observed that serum starvation of cardiac fibroblasts was a potent inhibitor of IL-1β release, while secretion of the inflammasome-independent cytokines TNF, MIP-2, and IL-6 was not attenuated (Figure 1A). Furthermore, LPS-induced pro-IL-1β mRNA was not affected by serum starvation (Figure 1B). LC3B is a well-established early marker of autophagy (21), typically featuring significant changes after 2–4 h of serum starvation (22). To investigate whether serum starvation induced autophagy in LPS-treated cardiac fibroblasts, the cells were incubated with 10% FCS or starved for 4 h with or without 10 ng/mL LPS before the amount of LC3B puncta were objectively quantified with high-throughput immune fluorescence microscopy (Figures 1C,D). In accordance with previous findings in macrophages (23), LPS alone was a significant inducer of autophagy in cardiac fibroblasts. However, no increase in autophagic activity could be observed after serum starvation, even in LPS-treated cells (Figure 1D).

### Serum Starvation Induces pro-IL-1β Degradation

**Figure 2:**
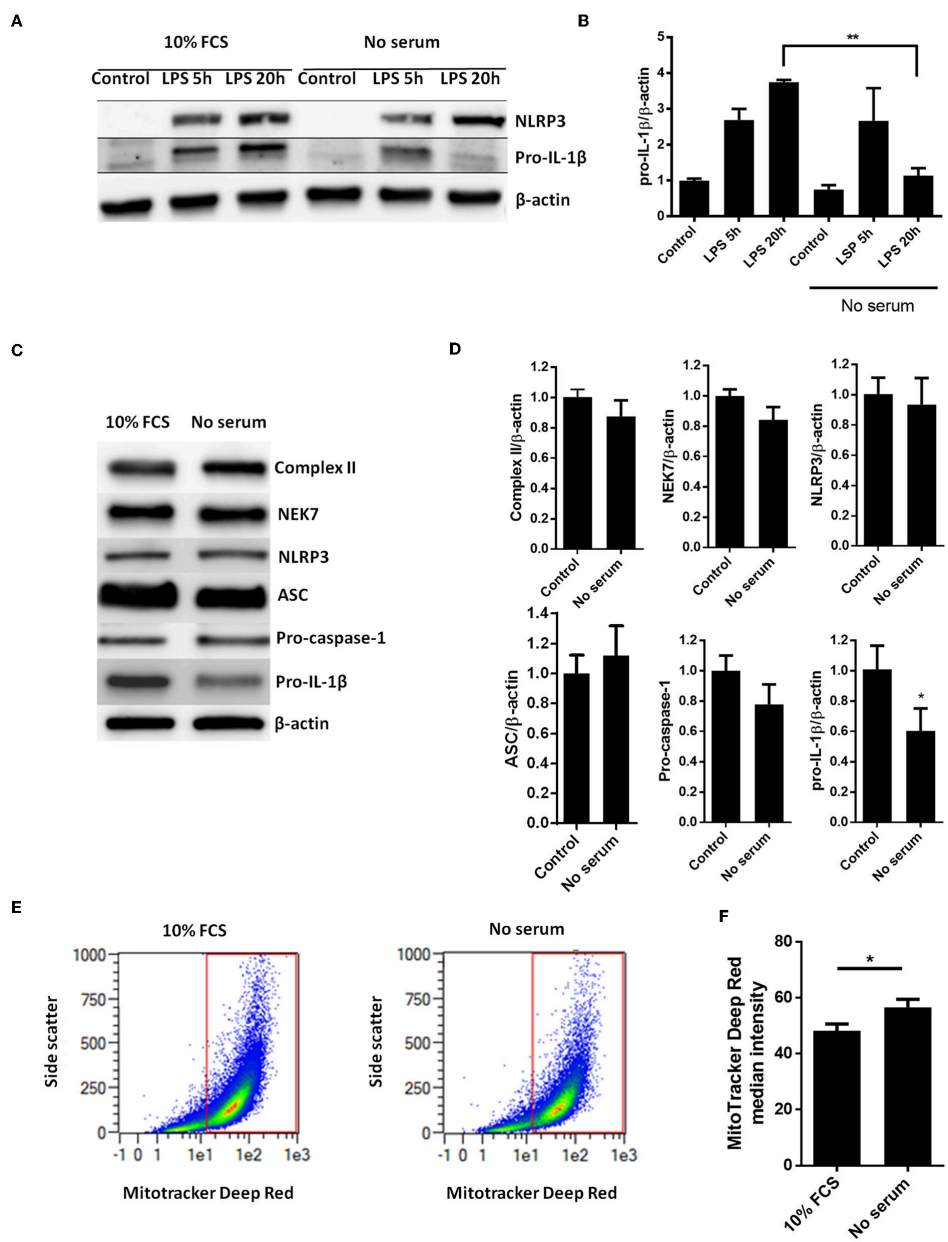
Serum starvation selectively induces pro-IL-1β degradation in cardiac fibroblasts. **(A)** Cells were incubated with 10 ng/mL LPS for 5 or 20 h with or without 10% FCS and western blot analysis of pro-IL-1β and NLRP3 performed. Blots are representative of 3 independent experiments. **(B)** Protein bands were quantified and the ratios to β-actin normalized to mean control = 1 (*n* = 3). **(C,D)** Cardiac fibroblasts were incubated with or without 10% FCS and primed with LPS for 20 h. Western blot analysis of complex II (mitochondrial mass marker) and NLRP3- inflammasome protein components (NEK7, NLRP3, ASC, pro-caspase-1, and pro-IL-1β) were performed. Bands were quantified and the ratios to β-actin normalized to mean control = 1 (*n* = 4, for ASC *n* = 10). **(E,F)** Cardiac fibroblasts (120,000 cells per well seeded in 6 well plates) were incubated with 10 ng/mL LPS for 20 h, with or without 10% FCS, then stained with 500 nM MitoTracker Deep Red for 45 min before MitoTracker fluorescence intensity was quantified with flow cytometry analysis (633 nm laser). All cells were analyzed and all gated cells included in the analysis. The presented dot plots **(E)** show the gate in red and are representative for 9 biological repeats. Median MitoTracker intensity with and without 10% FCS are shown **(F)**. All columns are mean with SEM. ^*^p < 0.05,^**^p < 0.01 (paired student *t*-test).

Whereas, LPS-induced pro-IL-1β mRNA was not affected by serum starvation (Figure 1B), serum starvation strongly downregulated the LPS-induced pro-IL-1β protein levels after 20 h of starvation (Figures 2A,B). We then quantified all known components of the NLRP3 inflammasome (NEK7, NLRP3, ASC, procaspase-1, and pro-IL-1β), as well as mitochondrial mass (complex II), in cardiac fibroblasts after 20 h of serum starvation (Figures 2C,D). Pro-IL-1β was downregulated while the expression of the other inflammasome proteins, as well as mitochondrial mass, was unaffected. Incidentally, ASC initially appeared to be downregulated. However, as opposed to pro-IL-1β, this finding was not reproduced in subsequent studies and pooled data are presented. Finally, we also investigated the effect of 20 h serum starvation on the mitochondrial mass by quantifying MitoTracker Deep Red fluorescence intensity in cardiac fibroblasts (Figures 2E,F). Serum starved cells featured significantly increased MitoTracker signaling. Together with western blot analysis of complex II, as well as indifferent LC3B puncta quantification, this strongly suggest that no mitophagy was induced by serum starvation in cardiac fibroblasts.

### The mTOR Inhibitor Rapamycin Rescues Pro-IL-1β From Serum Starvation Induced Degradation

**Figure 3:**
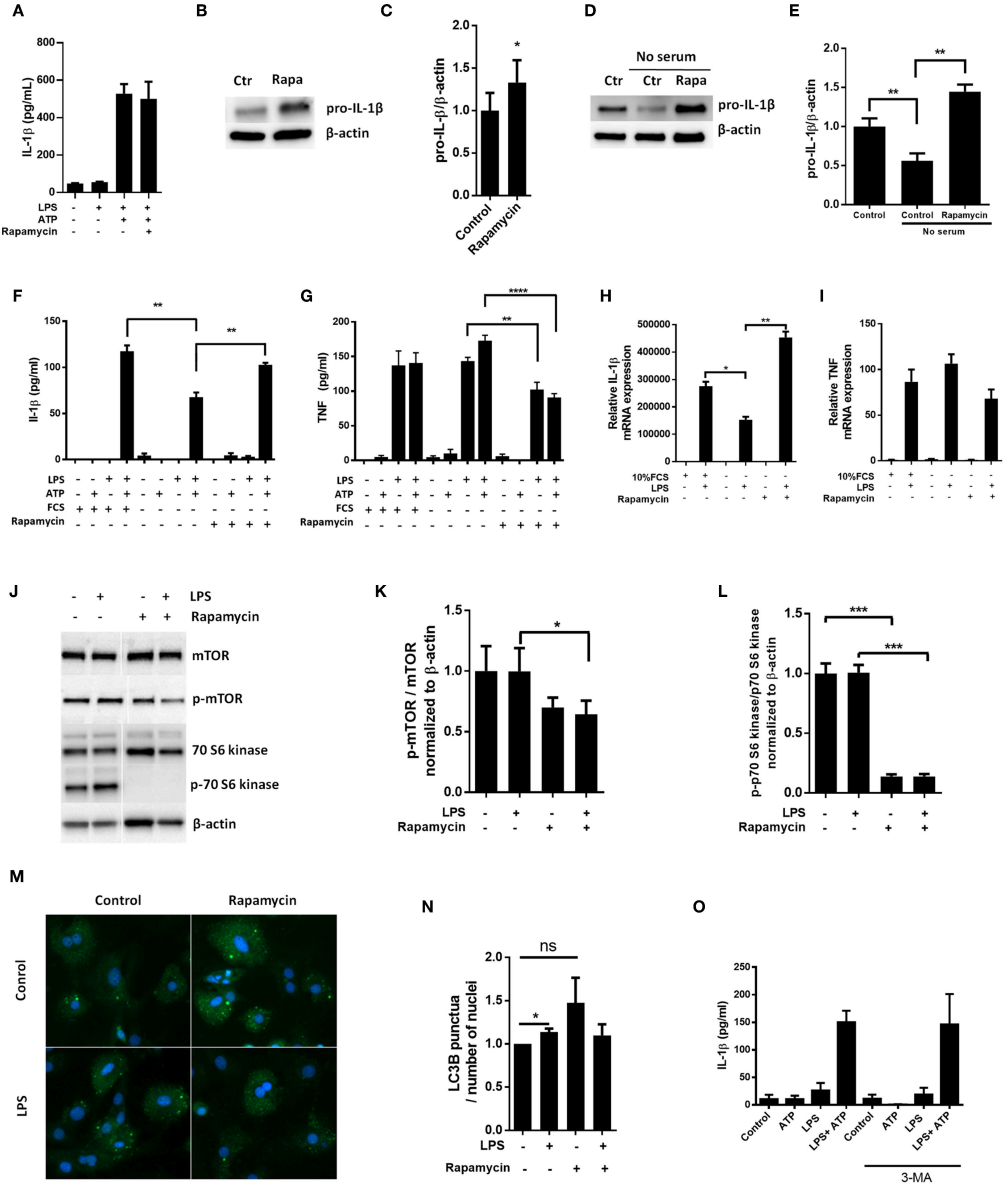
Rapamycin increases pro-IL-1β protein levels, rescues serum starvation-induced pro-IL-1β degradation, inhibits mTOR but induces no autophagy in LPS-stimulated cardiac fibroblasts. **(A)** IL-1β release was quantified in conditioned media from cardiac fibroblasts primed with 10 ng/mL LPS for 20 h with or without 500 nM rapamycin, as indicated, prior to activation with 3 mM ATP for 60 min. **(B,C)** Fibroblasts were primed with 10 ng/mL LPS for 20 h with or without 500 nM rapamycin and pro-IL-1β western blot performed (*n* = 3). (**D,E)** Control cells were incubated with 10% FCS, while serum deprived cells were incubated with or without 500 nM Rapamycin. Protein expressions were quantified with western blot (*n* = 4) **(F,G)**. IL-1β and TNF-α were quantified in conditioned media from cardiac fibroblasts incubated with or without 10% FCS and 500 nM rapamycin and stimulated with LPS 10 ng/mL for 20 h prior to activation with 3 mM ATP for 60 min as indicated (*n* = 6) (**H,I)** IL-1β and TNF mRNA were quantified with PCR. **(J–L)** Cardiac fibroblasts were incubated with 10% FCS with or without 10 ng/mL LPS and/or 500 nM rapamycin for 20 h and mTOR **(K)** and p70 S6 kinase **(L)** activity were quantified with western blot (*n* = 5). (**M,N)** Cardiac fibroblasts primed with LPS (10 ng/mL) were incubated with 10% FCS (control) or no serum for 4 h. Cells were labeled with anti-LC3B and Hoechst and whole slides scanned with an automated immunofluorescence microscope. LC3B puncta in all cells in were automatically counted and ratio to number of Hoechst-labeled kernels calculated. Columns are mean with SEM of paired data normalized to control = 1 (*n* = 5). **(O)** Cardiac fibroblasts were incubated with 10% FCS, 10 ng/mL LPS, and/or 5 mM 3-methyladenine (3-MA) for 18 h and/or ATP 3 mM for 60 min as indicated (*n* = 3). Rapa: rapamycin. 3-MA: 3-methyladenine, ns: not significant, ^*^p < 0.05, ^**^p < 0.01, ^****^p < 0.0001 (paired student *t*-test).

Pro-IL-1β has previously been reported to be a target for autophagy induced by rapamycin in macrophages (10). Hence, we sought to investigate if autophagy could have a role in pro-IL-1β degradation in cardiac fibroblasts by using rapamycin as a substitute for serum starvation. Surprisingly, rapamycin (500 nM) increased LPS-induced pro-IL-1β levels both with and without serum in the incubation media (Figures 3A–D). Indeed, rapamycin also protected pro-IL-1β from starvation-induced degradation and IL-1β secretion was restored with no significant difference compared to non-starved cells (Figures 3D–F). Furthermore, rapamycin significantly increased pro-IL-1β mRNA levels (Figure 3H). In accordance with previous publications (24–26), rapamycin significantly decreased TNF release (Figure 3G). However, no significant change in TNF mRNA was observed (Figure 3I), contradicting a previous report of mRNA destabilization as the plausible mechanism (25). Although rapamycin effectively inhibited mTOR and its downstream p70 S6 kinase activity (Figures 3J–L), it did not induce autophagy in LPS stimulated cardiac fibroblasts as compared to LPS-treated control cells (Figures 3M,N). In accordance with this, 3-methyladenine (3-MA), another promoter of autophagy under non-starving conditions (27), had no effect on IL-1β release from cardiac fibroblasts (Figure 3O). Of note, rapamycin has previously been reported to promote apoptosis in high doses (0.2–20 μM) (28), in which case cleaved caspase-8 could contribute to IL-1β release (29). However, we observed no evidence of caspase-8 activation in cardiac fibroblasts with western blot analysis (Supplementary Figure 2).

### The Autophagy Inhibitors Chloroquine and Bafilomycin A1 Induce Pro-IL-1β Degradation

**Figure 4:**
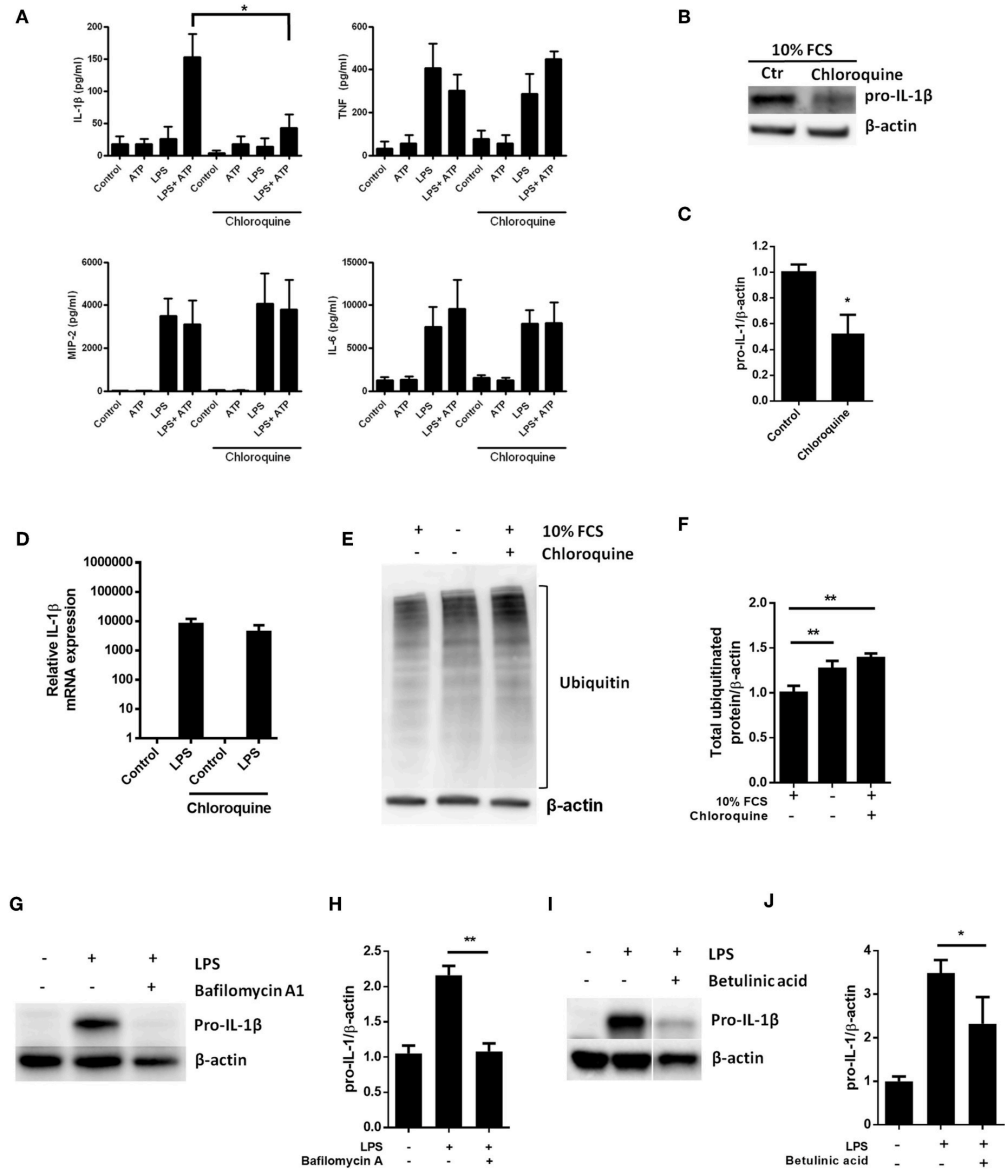
Pro-IL-1β degradation is induced by the autophagy inhibitors chloroquine and bafilomycin A, and by the proteasome activator betulinic acid. **(A)** Chloroquine selectively attenuates IL-1β release from cardiac fibroblasts. The cells were incubated with LPS and/or 20 μM chloroquine phosphate as indicated for 18 h, then activated with 3 mM ATP for 60 min. IL-1β, TNF, MIP-2, and IL-6 were quantified in the conditioned media (*n* = 3). (**B,C)** Cardiac fibroblasts were incubated with LPS for 18 h with or without 20 μM chloroquine phosphate and pro-IL-1β quantified with western blot (*n* = 3). **(D)** Pro-IL-1β mRNA expression was quantified with qPCR (*n* = 3). (**E,F)** Cardiac fibroblasts were incubated with LPS for 20 h with or without 10% FCS or 20 μM chloroquine phosphate as indicated. The amount of total ubiquitinated protein was quantified with western blot (*n* = 4). (**G,H)** Cardiac fibroblasts were incubated with 10% FCS with or without 10 ng/mL LPS and/or 100 nM bafilomycin A1 for 20 h. Pro-IL-1β was quantified with western blot. **(I,J)** Cardiac fibroblasts were incubated with 10% FCS with or without 10 ng/mL LPS and/or 20 μM betulinic acid as indicated for 20 h. Pro-IL-1β was quantified with western blot. All columns are mean with SEM. ^*^p < 0.05, ^**^p < 0.01 (paired student *t*-test).

LPS-stimulation has been reported to increase baseline autophagy in macrophages and even cardiomyocytes (23, 30), suggesting that TLR4 signaling is a general autophagy inducer. Indeed, this is in line with our current data showing LPS-induced autophagy in cardiac fibroblasts (Figure 1D). Thus, given a role of autophagy in pro-IL-1β degradation, inhibiting autophagy pharmacologically could increase LPS-induced pro-IL-1β protein levels. Chloroquine potently inhibits autophagy by impairing autophagosome fusion with lysosomes (31). However, chloroquine significantly reduced IL-1β release from ATP-activated LPS-primed cardiac fibroblasts while LPS-induced secretion of TNF, MIP-2, and IL-6 were not affected (Figure 4A). Furthermore, chloroquine reduced LPS-induced pro-IL-1β protein levels (Figures 4B,C) without affecting IL-1β mRNA expression levels (Figure 4D). By inhibiting autophagy, however, chloroquine has been reported to increase proteasomal activity (32, 33). Indeed, both serum starvation and chloroquine significantly increased total protein ubiquitination, indicating increased proteasomal activity (Figures 4E,F). Furthermore, bafilomycin A1, which also inhibits autophagy by preventing fusion between autophagosomes and lysosomes (34), potently reduced pro-IL-1β protein levels (Figures 4G,H). Finally, the proteasome activator betulinic acid significantly reduced pro-IL-1β levels and attenuated IL-1β release (Figures 4I,J). Thus, our data suggest that chloroquine and bafilomycin A1 induce pro-IL-1β protein degradation through proteasomal degradation.

### Chloroquine Induces Pro-IL-1β Degradation in Human Macrophages and Attenuates IL-1β Release

**Figure 5:**
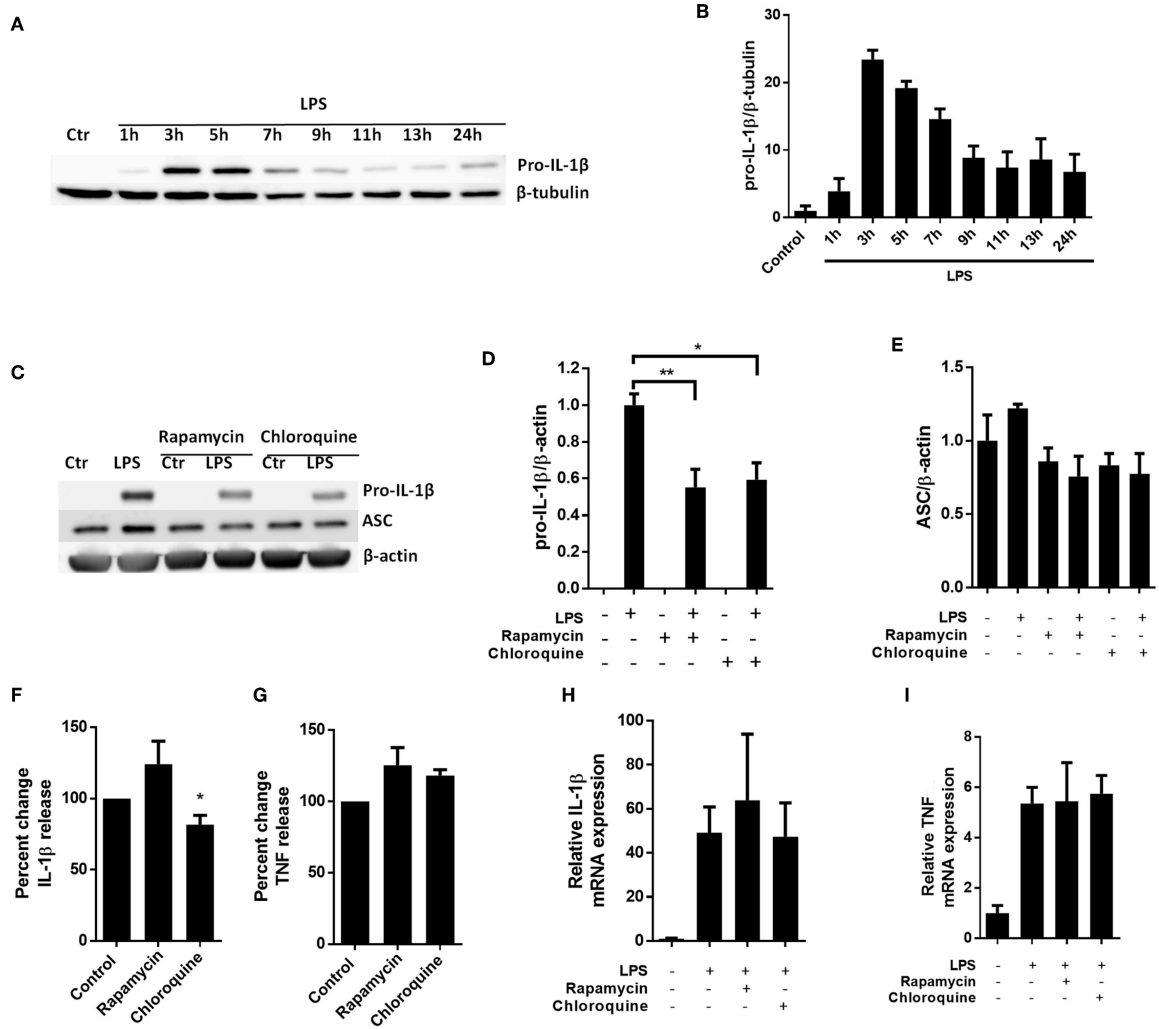
Chloroquine but not rapamycin attenuates IL-1β release from monocyte-derived M-CSF-differentiated human macrophages. (**A,B)** Macrophages were stimulated with 10 ng/mL LPS for times as indicated and pro**-**IL-1β was quantified with western blot. **(C–E)** Macrophages were incubated with 10 ng/mL LPS for 5 h with or without 500 nM rapamycin or 20 μM chloroquine and pro-IL-β and ASC quantified with western blot (*n* = 3). Columns are mean with SEM. **(F,G)** Macrophages were incubated with 10 ng/mL LPS for 5 h with or without 500 nM rapamycin or 20 μM chloroquine phosphate, then 3 mM ATP for 30 min. IL-1β and TNF release were quantified in conditioned medium with ELISA and percent change in output calculated. Data are normalized to control = 100%. **(H,I)** Macrophages were incubated with 10 ng/mL LPS for 5 h with or without 500 nM rapamycin or 20 μM chloroquine phosphate. IL-1β and TNF mRNA was quantified with PCR. All columns are mean with SEM (*n* = 3–6). ^*^p < 0.05, ^**^p < 0.01 as compared to control or as indicated (paired student *t*-test).

To investigate whether autophagy-independent pro-IL-1β degradation could be a general mechanism for regulating IL-1β signaling, we sought to reproduce key findings in primary human macrophages (Figure 5). Interestingly and as opposed to cardiac fibroblasts, the macrophages spontaneously started to degrade the LPS-induced pro-IL-1β if the cells were incubated with LPS for more than 5 h (Figures 5A,B). Both rapamycin and chloroquine significantly reduced pro-IL-1β protein levels while ASC was not affected (Figures 5C,D). However, only for chloroquine did this translate to reduced IL-1β secretion after activation of the NLRP3 inflammasome with ATP (Figures 5F,G). Again, pro-IL-1β and TNF mRNA levels were not affected by these interventions (Figures 5H,I).

## Discussion

In this study we hypothesized that autophagy could serve as an anti-inflammatory regulatory mechanism of NLRP3-dependent IL-1β-release in cardiac fibroblasts. Serum starvation selectively decreased pro-IL-1β protein expression and attenuated IL-1β secretion. Although LPS increased baseline autophagy in accordance with previous reports, we were unable to induce autophagy with serum starvation or rapamycin in cardiac fibroblasts. Furthermore, LPS-induced pro-IL-1β and subsequent ATP-induced IL-1β-release were rescued by the mTOR inhibitor rapamycin in serum starved cells without affecting autophagy. Finally, autophagy inhibition with chloroquine reproduced pro-IL-1β degradation and attenuated IL-1β release from cardiac fibroblasts. Hence, our data indicated that IL-1β may be removed by proteasomal degradation. This hypothesis is further supported by our observations that: (1) chloroquine increases total protein ubiquitination; (2) the effect of chloroquine can be reproduced by bafilomycin A1, a pharmacodynamical similar autophagy inhibitor; (3) the proteasome activator betulinic acid induces pro-IL-1β degradation. Our findings thus that suggest mTOR and proteasome activation, not autophagy, attenuate pro-IL-1β levels and IL-1β secretion from cardiac fibroblasts.

If cardiac fibroblasts only receives signal 1, pro-IL-1β, as well as inflammasome components, are synthesized but no IL-1β is released (9). In human macrophages *in vitro*, pro-IL-1β is degraded if the second signal is not given within about 10 h (Figures 5A,B). In cardiac fibroblasts, however, pro-IL-1β persists, as shown in this study and previously (9). In relation to the myocardium, signal 1 may be delivered due to a leaky gut barrier (35) or chronic inflammation originating elsewhere in the organism (36). In that case, inducing pro-IL-1β degradation in cardiac cells may limit subsequent inflammatory responses to cardiac tissue damage and hence serve as a cardio protective prophylactic intervention in patients with ischemic heart disease or heart failure. Interestingly, Liu et al. recently published a meta-analysis suggesting that chloroquine reduces the risk of cardiovascular disease in patients with rheumatic disease (37). Moreover, in a prospective placebo controlled trial, Hartman et al. will investigate if chloroquine can prevent new cardiovascular events in patients who have already suffered a myocardial infarction (38). Our current study suggest that IL-1β may be implicated in any cardioprotective feature of chloroquine. To address such protective mechanisms of chloroquine on infarct size *in vivo*, however, a cardiac ischemia-reperfusion model of mice pre-treated with chloroquine may be the next logic step.

We have investigated the effect of serum starvation on the expression of all proteins known to be directly involved in NLRP3-dependent IL-1β-release in general (NEK7, NLRP3, ASC, procaspase-1, and pro-IL-1β), excluding proteins only relevant to specific danger signal pathways. We also quantified mitochondrial mass by the expression of complex II and MitoTracker. However, only pro-IL-1β protein levels were significantly and consistently reduced in all subsequent experiments including serum starvation. Thus, our findings suggest that the effect of serum starvation on IL-1β release seems to reflect a selective effect on pro-IL-1β degradation and no other inflammasome proteins.

Several studies suggest a role for autophagy in regulating IL-1β release from innate immune cells. Data supporting this hypothesis have been obtained from experiments with pharmacological inhibitors and inducers of autophagy (10, 39), as well as with cells carrying genetic loss of autophagic function (40). Autophagy can also be induced by amino-acid deprivation or growth factor withdrawal (20, 41). However, herein we showed that serum starvation reduced pro-IL-1β protein levels while no increase in autophagy could be observed. mTOR is a key regulator of autophagy, and to further investigate our original hypothesis we then sought to use a pharmacological inducer of autophagy, the mTOR inhibitor rapamycin. In 2011, Harris et al. showed that rapamycin induced autophagy in mouse bone marrow-derived macrophages, resulting in significantly reduced pro-IL-1β levels and IL-1β secretion (10). However, relatively high doses of rapamycin were used in that study (12.5–50 μg/mL = 13.1–54.7 μM). On the other hand, in 2016 Sotthibundhu et al. observed that 200 nM was an optimal dose for induction of autophagy in induced pluripotent stem cells (42), 300 nM was less efficient. In our study, 500 nM of rapamycin effectively inhibited mTOR activity but induced no increase in autophagy in LPS-stimulated cardiac fibroblasts compared to LPS-stimulated control cells. Surprisingly, rapamycin had a profound effect of increasing pro-IL-1β mRNA and protein levels, even rescuing IL-1β secretion from serum starved cells. Thus, mTOR inhibition favors LPS-induced pro-IL-1β but not TNF synthesis in cardiac fibroblasts. Our findings thus suggest that mTOR could be of importance for downregulating pro-IL-1β levels and secretion in cardiac fibroblast during serum starvation. In contrast, we observed that rapamycin decreased pro-IL-1β protein levels in monocyte-derived human macrophages, which is in line with the findings of Harris et al. (10). Thus, our data do not support an overall pro-inflammatory effect of rapamycin *in vivo*, merely that mTOR is implicated in pro-IL-1β catabolism in cardiac fibroblasts. Finally, we have not investigated the effect of increased mTOR activation, such as through mTOR overexpression, on pro-IL-1β expression in cardiac fibroblasts. This may weaken our findings. Interestingly, rapamycin has been shown to be a proteasome inhibitor, inhibiting PA28 mRNA and protein expression (43) at 50 nM, as well as allosterically inhibiting the proteolytic activity of the 20S proteasome, Osmulski et al. (44) and it is tempting to hypothesize that the enhancing effect of mTOR inhibition on pro-IL-1β levels could also reflect proteasome inhibition, and thereby attenuated pro-IL-1β degradation.

Since TLR4 signaling has been reported to increase baseline autophagy (23, 30), IL-1β secretion could potentially be increased by inhibiting autophagy, given that pro-IL-1β indeed is an autophagy target, specifically or in general. The cardiac fibroblasts were incubated with LPS (signal 1) for 18-20 h prior to ATP activation (signal 2), leaving plenty of time for pro-IL-1β removal. However, increasing the pro-IL-1β pool, as well as IL-1β secretion, with chloroquine failed completely. Chloroquine and bafilomycin A1 both inhibit autophagy by impairing autophagosome fusion with lysosomes (31). This then, increases proteasome activity (32, 33). Proteasome inhibitors, such as MG-132, could counter the effect of chloroquine and bafilomycin A1 on pro-IL-1β protein levels. The lack of such data weakens our findings. However, we do show that a proteasome activator, betulinic acid, is sufficient to decrease pro-IL-1β levels and IL-1β release. Interestingly, in 2015, Bourke et al. showed that pretreatment of rats with chloroquine for 3 days significantly reduced myocardial infarct size after 1 h ischemia followed by a 24 h reperfusion period (45). IL-1 signaling was not addressed, however. More recently, Chen et al. reported that chloroquine reduced NLRP3-dependent IL-1β release from mouse bone marrow derived macrophages (46). However, their main mechanistic finding was that chloroquine inhibited LPS-induced NF-κB activation (signal 1), leading to reduced pro-IL-1β and NLRP3 mRNA synthesis. In contrast, our current data shows that chloroquine induced pro-IL-1β protein degradation without affecting pro-IL-1β mRNA. Furthermore, we repeated our experiments addressing the effects of rapamycin and chloroquine on NLRP3-dependent IL-1β secretion on primary human monocyte-derived macrophages. Again, we found that chloroquine reduced pro-IL-1β protein levels and attenuated IL-1β release, while TNF release, IL-1β mRNA, and TNF mRNA were not affected. Hence, our data do not support any interference of chloroquine with signal 1, but strongly suggest that chloroquine induces proteasomal degradation of pro-IL-1β protein.

**Figure 6:**
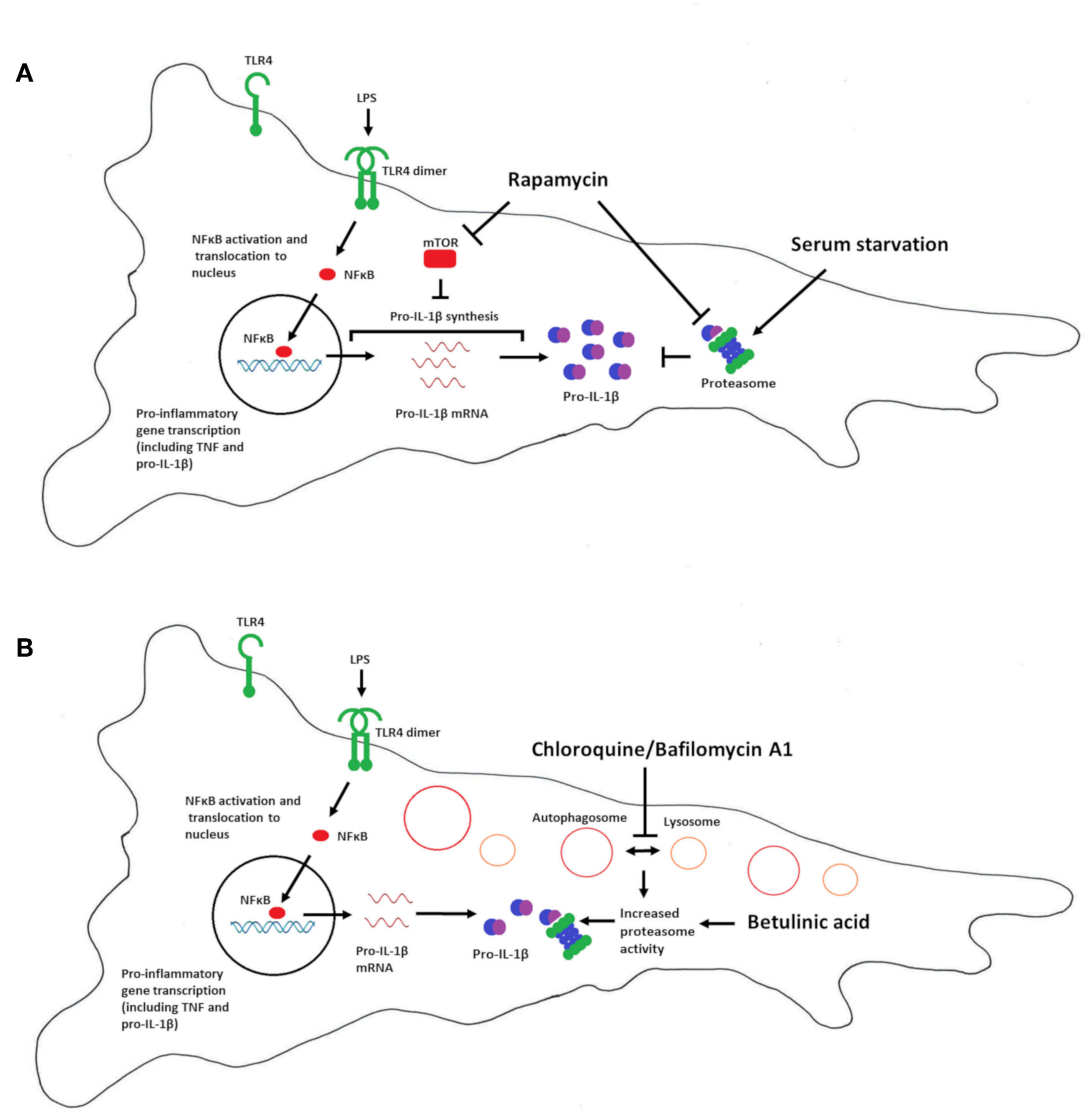
Summary of the effects of serum starvation, rapamycin, chloroquine, bafilomycin A1 and betulinic acid on pro-IL-1β expression in cardiac fibroblasts. **(A)** Serum starvation (right side) increases proteasome activity and pro-IL-1β protein degradation. mTOR inhibition with rapamycin favors LPS-induced pro-IL-1β synthesis, reflected by increased pro-IL-1β mRNA and protein levels. Moreover, rapamycin has been reported to inhibit proteasome activity by inhibiting PA28 mRNA and protein expression (43), as well as allosterically inhibiting the proteolytic activity of the 20S proteasome (44), which may further contribute to increased pro-IL-1β protein levels. Rapamycin thus restores LPS-induced pro-IL-1β levels in serum-starved cells. **(B)** Chloroquine and bafilomycin A1 inhibits autophagy by preventing the fusion of lysosomes with autophagosomes (which then accumulates in the cell). Lack of autophagy increases proteasomal activity, leading to degradation of pro-IL-1β protein (33). Direct activation of the proteasome with betulinic acid also results in pro-IL-1β protein degradation.

Figure 6 summarizes our interpretation of the data presented in this study. Our data imply that mTOR is a negative regulator of pro-IL-1β synthesis in cardiac fibroblasts, while proteasomal degradation of pro-IL-1β, and not autophagy, is the major catabolic anti-inflammatory mechanism regulating this cytokine. We propose that proteasomal degradation of pro-IL-1β may contribute to the anti-inflammatory effects of chloroquine as well as caloric restriction.

## Ethics Statement

The part of the study that included human monocytes was approved by the local ethical committee (Regional ethics committee of Helse Sør; Øst; Permit number S-05172) and conducted according to the ethical guidelines outlined in the Declaration of Helsinki for use of human tissue and subjects. Animal experiments (isolation of primary mouse cardiac fibroblasts) were approved by the Norwegian Animal Research Authority 80 project license no FOTS id 7,333 and 13,643. The isolations of primary animal cells were performed in accordance with the European Directive 2010/63/EU and The Guide for the Care and Use of Laboratory Animals, 8th edition (NRC 2011, National Academic Press).

## Author Contributions

M-KT has performed a major part of the experiments and analysis in relation to cardiac fibroblasts. M-KT also isolated and cultured cardiac fibroblasts together with TR. KY has isolated monocytes, differentiated macrophages, and performed all experiments and analysis in relation to macrophages. TR isolated cardiac fibroblasts together with M-KT. TR also performed flow cytometry analysis and the associated experiments. KL performed western blot analysis. KA cultured cardiac fibroblasts isolated by LV in the initial phase of this study. She also performed ELISA analysis. LV isolated cardiac fibroblasts in the initial phase of this study. PA and K-OS co-supervised the study and have performed major contribution to data interpretation and manuscript writing. AY was the main supervisor in the initial phase. He developed the original hypothesis and has performed major contribution to data interpretation and manuscript writing. ØS has performed the experiments and analysis in relation to cardiac fibroblasts in the initial phase and has written the initial manuscript. He has been the main supervisor in the late phase of this study. All authors have contributed to the writing of the final manuscript.

### Conflict of Interest Statement

The authors declare that the research was conducted in the absence of any commercial or financial relationships that could be construed as a potential conflict of interest.

## Supplementary Material

Supplementary Figure 1 A role for NEK7 and mitochondrial ROS in NLRP3-mediated IL-1β release in mouse cardiac fibroblasts. **(A)** NEK7 co-localizes with ASC after NLRP3-dependent inflammasome formation. Cardiac fibroblasts were primed with 10 ng/mL LPS for 18 h, then activated with 3 mM ATP for 60 min. The cells were fixed and labeled with anti-ASC Alexa 633 and anti-NEK7 Alexa488. **(B)** The mitochondria specific ROS scavenger MitoTempo inhibits IL-1β release, while TNF-α is not affected. Cardiac fibroblasts were incubated in medium only or 10 ng/ml LPS for 20 h, then mtROS scavenger MitoTempo (100 uM) for 60 min prior to ATP (3 mM) for 60 min. Columns are mean with SEM (*n* = 3).

Supplementary Figure 2 No role for caspase-8 in IL-1β release from serum starved cardiac fibroblasts treated with LPS and rapamycin. Cardiac fibroblasts were incubated with or without 10% FCS and primed with 10 ng/mL LPS with or without rapamycin (500 nM) for 20 h prior to activation with ATP for 60 min. Western blot analysis of cleaved caspase-8 were performed. HL-1 cells treated with 10 μM staurosporine for 3 h followed by 22 h incubation in staurosproine-free medium served as positive control. Blot is representative for six independent biological repeats.

## References

[1] Broz P Dixit VM. Inflammasomes: mechanism of assembly, regulation, and signalling. Nat Rev Immunol. (2016) 16:407–20. 10.1038/nri.2016.58 27291964

[2] Schmid-Burgk JL Chauhan D Schmidt T Ebert TS Reinhardt J Endl E. A Genome-wide CRISPR (Clustered Regularly Interspaced Short Palindromic Repeats) screen identifies NEK7 as an essential component of NLRP3 inflammasome activation. J Biol Chem. (2016) 291:103–9. 10.1074/jbc.C115.700492 26553871 PMC4697147

[3] He Y Zeng MY Yang D Motro B Núñez G. NEK7 is an essential mediator of NLRP3 activation downstream of potassium efflux. Nature. (2016) 530:354–7. 10.1038/nature16959 26814970 PMC4810788

[4] Shi H Wang Y Li X Zhan X Tang M Fina M. NLRP3 activation and mitosis are mutually exclusive events coordinated by NEK7, a new inflammasome component. Nat Immunol. (2016) 17:250–8. 10.1038/ni.3333 26642356 PMC4862588

[5] Sager HB Heidt T Hulsmans M Dutta P Courties G Sebas M. Targeting interleukin-1beta reduces leukocyte production after acute myocardial infarction. Circulation. (2015) 132:1880–90. 10.1161/CIRCULATIONAHA.115.016160 26358260 PMC4651795

[6] Guillén I Blanes M Gómez-Lechón MJ Castell JV. Cytokine signaling during myocardial infarction: sequential appearance of IL-1 beta and IL-6. Am J Physiol. (1995) 269:R229–35. 10.1152/ajpregu.1995.269.2.R229 7544543

[7] Bujak M Dobaczewski M Chatila K Mendoza LH Li N Reddy A. Interleukin-1 receptor type I signaling critically regulates infarct healing and cardiac remodeling. Am J Pathol. (2008) 173:57–67. 10.2353/ajpath.2008.070974 18535174 PMC2438285

[8] Abbate A Van Tassell BW Biondi-Zoccai G Kontos MC Grizzard JD Spillman DW. Effects of interleukin-1 blockade with anakinra on adverse cardiac remodeling and heart failure after acute myocardial infarction [from the Virginia Commonwealth University-Anakinra Remodeling Trial (2) (VCU-ART2) pilot study]. Am J Cardiol. (2013) 111:1394–400. 10.1016/j.amjcard.2013.01.287 23453459 PMC3644511

[9] Sandanger Ø Ranheim T Vinge LE Bliksøen M Alfsnes K Finsen AV. The NLRP3 inflammasome is up-regulated in cardiac fibroblasts and mediates myocardial ischaemia-reperfusion injury. Cardiovasc Res. (2013) 99:164–74. 10.1093/cvr/cvt091 23580606

[10] Harris J Hartman M Roche C Zeng SG O'Shea A Sharp FA. Autophagy controls IL-1beta secretion by targeting pro-IL-1beta for degradation. J Biol Chem. (2011) 286:9587–97. 10.1074/jbc.M110.202911 21228274 PMC3058966

[11] Nakahira K Haspel JA Rathinam VA Lee SJ Dolinay T Lam HC. Autophagy proteins regulate innate immune responses by inhibiting the release of mitochondrial DNA mediated by the NALP3 inflammasome. Nat Immunol. (2011) 12:222–30. 10.1038/ni.1980 21151103 PMC3079381

[12] Zhao J Garcia GA Goldberg AL. Control of proteasomal proteolysis by mTOR. Nature. (2016) 529:E1–2. 10.1038/nature16472 26791731 PMC4765347

[13] Nazio F Strappazzon F Antonioli M Bielli P Cianfanelli V Bordi M. mTOR inhibits autophagy by controlling ULK1 ubiquitylation, self-association and function through AMBRA1 and TRAF6. Nat Cell Biol. (2013) 15:406–16. 10.1038/ncb2708 23524951

[14] Howell JJ Ricoult SJ Ben-Sahra I Manning BD. A growing role for mTOR in promoting anabolic metabolism. Biochem Soc Trans. (2013) 41:906–12. 10.1042/BST20130041 23863154

[15] Jung CH Ro SH Cao J Otto NM Kim DH. mTOR regulation of autophagy. FEBS Lett. (2010) 584:1287–95. 10.1016/j.febslet.2010.01.017 20083114 PMC2846630

[16] Kamentsky L Jones TR Fraser A Bray MA Logan DJ Madden KL. Improved structure, function and compatibility for CellProfiler: modular high-throughput image analysis software. Bioinformatics. (2011) 27:1179–80. 10.1093/bioinformatics/btr095 21349861 PMC3072555

[17] Misawa T Takahama M Kozaki T Lee H Zou J Saitoh T. Microtubule-driven spatial arrangement of mitochondria promotes activation of the NLRP3 inflammasome. Nat Immunol. (2013) 14:454–60. 10.1038/ni.2550 23502856

[18] Wang K Yao Y Zhu X Zhang K Zhou F Zhu L. Amyloid beta induces NLRP3 inflammasome activation in retinal pigment epithelial cells via NADPH oxidase- and mitochondria-dependent ROS production. J Biochem Mol Toxicol. (2017) 31:e21887. 10.1002/jbt.21887 28004443

[19] Zhou R Yazdi AS Menu P Tschopp J. A role for mitochondria in NLRP3 inflammasome activation. Nature. (2011) 469:221–5. 10.1038/nature09663 21124315

[20] Lee Y Lee HY Gustafsson AB. Regulation of autophagy by metabolic and stress signaling pathways in the heart. J Cardiovasc Pharmacol. (2012) 60:118–24. 10.1097/FJC.0b013e318256cdd0 22472907 PMC3419780

[21] Tanida I Ueno T Kominami E. LC3 and Autophagy. Methods Mol Biol. (2008) 445:77–88. 10.1007/978-1-59745-157-4_4 18425443

[22] Zhang Z Singh R Aschner M. Methods for the detection of autophagy in mammalian cells. Curr Protoc Toxicol. (2016) 69:20.12.1-20.12.26. 10.1002/cptx.11 27479363 PMC4982470

[23] Yuan H Perry CN Huang C Iwai-Kanai E Carreira RS Glembotski CC. LPS-induced autophagy is mediated by oxidative signaling in cardiomyocytes and is associated with cytoprotection. Am J Physiol Heart Circ Physiol. (2009) 296:H470–9. 10.1152/ajpheart.01051.2008 19098111 PMC2643899

[24] Yard BA Pancham RR Paape ME Daha MR van Es LA van der Woude FJ. CsA, FK506, corticosteroids and rapamycin inhibit TNF alpha production by cultured PTEC1. Kidney Int. (1993) 44:352–8. 10.1038/ki.1993.251 8377379

[25] Park JW Jeon YJ Lee JC Ahn SR Ha SW Bang SY. Destabilization of TNF-alpha mRNA by Rapamycin. Biomol Ther. (2012) 20:43–9. 10.4062/biomolther.2012.20.1.043 24116273 PMC3792200

[26] Lorne E Zhao X Zmijewski JW Liu G Park YJ Tsuruta Y. Participation of mammalian target of rapamycin complex 1 in Toll-like receptor 2- and 4-induced neutrophil activation and acute lung injury. Am J Respir Cell Mol Biol. (2009) 41:237–45. 10.1165/rcmb.2008-0290OC 19131641 PMC2715911

[27] Wu YT Tan HL Shui G Bauvy C Huang Q Wenk MR. Dual role of 3-methyladenine in modulation of autophagy via different temporal patterns of inhibition on class I and III phosphoinositide 3-kinase. J Biol Chem. (2010) 285:10850–61. 10.1074/jbc.M109.080796 20123989 PMC2856291

[28] Castedo M Ferri KF Kroemer G. Mammalian target of rapamycin (mTOR): pro- and anti-apoptotic. Cell Death Differ. (2002) 9:99–100. 10.1038/sj.cdd.4400978 11840159

[29] England H Summersgill HR Edye ME Rothwell NJ Brough D. Release of interleukin-1alpha or interleukin-1beta depends on mechanism of cell death. J Biol Chem. (2014) 289:15942–50. 10.1074/jbc.M114.557561 24790078 PMC4047367

[30] Xu Y Jagannath C Liu XD Sharafkhaneh A Kolodziejska KE Eissa NT. Toll-like receptor 4 is a sensor for autophagy associated with innate immunity. Immunity. (2007) 27:135–44. 10.1016/j.immuni.2007.05.022 17658277 PMC2680670

[31] Mauthe M Orhon I Rocchi C Zhou X Luhr M Hijlkema KJ. Chloroquine inhibits autophagic flux by decreasing autophagosome-lysosome fusion. Autophagy. (2018) 14:1435–55. 10.1080/15548627.2018.1474314 29940786 PMC6103682

[32] Kimura N Kumamoto T Oniki T Nomura M Nakamura K Abe Y. Role of ubiquitin-proteasome proteolysis in muscle fiber destruction in experimental chloroquine-induced myopathy. Muscle Nerve. (2009) 39:521–8. 10.1002/mus.21223 19296457

[33] Wang XJ Yu J Wong SH Cheng AS Chan FK Ng SS. A novel crosstalk between two major protein degradation systems: regulation of proteasomal activity by autophagy. Autophagy. (2013) 9:1500–8. 10.4161/auto.25573 23934082

[34] Yamamoto A Tagawa Y Yoshimori T Moriyama Y Masaki R Tashiro Y. Bafilomycin A1 prevents maturation of autophagic vacuoles by inhibiting fusion between autophagosomes and lysosomes in rat hepatoma cell line, H-4-II-E cells. Cell Struct Funct. (1998) 23:33–42. 10.1247/csf.23.33 9639028

[35] Ebner N Földes G Schomburg L Renko K Springer J Jankowska EA. Lipopolysaccharide responsiveness is an independent predictor of death in patients with chronic heart failure. J Mol Cell Cardiol. (2015) 87:48–53. 10.1016/j.yjmcc.2015.07.029 26264758

[36] Løgstrup BB Ellingsen T Pedersen AB Kjærsgaard A Bøtker HE Maeng M. Heart failure and ischemic heart disease in patients with rheumatoid arthritis. J Am Coll Cardiol. (2017) 70:3069–71. 10.1016/j.jacc.2017.10.028 29241494

[37] Liu D Li X Zhang Y Kwong JS Li L Zhang Y. Chloroquine and hydroxychloroquine are associated with reduced cardiovascular risk: a systematic review and meta-analysis. Drug Des Devel Ther. (2018) 12:1685–95. 10.2147/DDDT.S166893 29928112 PMC6001837

[38] Hartman O Kovanen PT Lehtonen J Eklund KK Sinisalo J. Hydroxychloroquine for the prevention of recurrent cardiovascular events in myocardial infarction patients: rationale and design of the OXI trial. Eur Heart J Cardiovasc Pharmacother. (2017) 3:92–7. 10.1093/ehjcvp/pvw035 28025216

[39] Crişan TO Plantinga TS van de Veerdonk FL Farcaş MF Stoffels M Kullberg BJ. Inflammasome-independent modulation of cytokine response by autophagy in human cells. PLoS ONE. (2011) 6:e18666. 10.1371/journal.pone.0018666 21490934 PMC3072416

[40] Saitoh T Fujita N Jang MH Uematsu S Yang BG Satoh T. Loss of the autophagy protein Atg16L1 enhances endotoxin-induced IL-1beta production. Nature. (2008) 456:264–8. 10.1038/nature07383 18849965

[41] Harris J. Autophagy and IL-1 family cytokines. Front Immunol. (2013) 4:83. 10.3389/fimmu.2013.00083 23577011 PMC3617358

[42] Sotthibundhu A McDonagh K von Kriegsheim A Garcia-Munoz A Klawiter A Thompson K. Rapamycin regulates autophagy and cell adhesion in induced pluripotent stem cells. Stem Cell Res Ther. (2016) 7:166. 10.1186/s13287-016-0425-x 27846905 PMC5109678

[43] Wang X Omura S Szweda LI Yang Y Bérard J Seminaro J. Rapamycin inhibits proteasome activator expression and proteasome activity. Eur J Immunol. (1997) 27:2781–6. 10.1002/eji.1830271106 9394799

[44] Osmulski PA Gaczynska M Osmulski PA Gaczynska M. Rapamycin allosterically inhibits the proteasome. Mol Pharmacol. (2013) 84:104–13. 10.1124/mol.112.083873 23619386

[45] Bourke L McCormick J Taylor V Pericleous C Blanchet B Costedoat-Chalumeau N. Hydroxychloroquine protects against cardiac ischaemia/reperfusion injury *in vivo* via enhancement of ERK1/2 phosphorylation. PLoS ONE. (2015) 10:e0143771. 10.1371/journal.pone.0143771 26636577 PMC4670100

[46] Chen X Wang N Zhu Y Lu Y Liu X Zheng J. The antimalarial chloroquine suppresses LPS-induced NLRP3 inflammasome activation and confers protection against murine endotoxic shock. Mediators Inflamm. (2017) 2017:6543237. 10.1155/2017/6543237 28321151 PMC5340938

