# Supplementary figures and images for: Mammalian Target of Rapamycin (mTOR) and the Proteasome Attenuates IL-1β Expression in Primary Mouse Cardiac Fibroblasts
Source: Front Immunol. 2019 Jun 6;10:1285. doi: 10.3389/fimmu.2019.01285 (PMC6563870; doi:10.3389/fimmu.2019.01285)

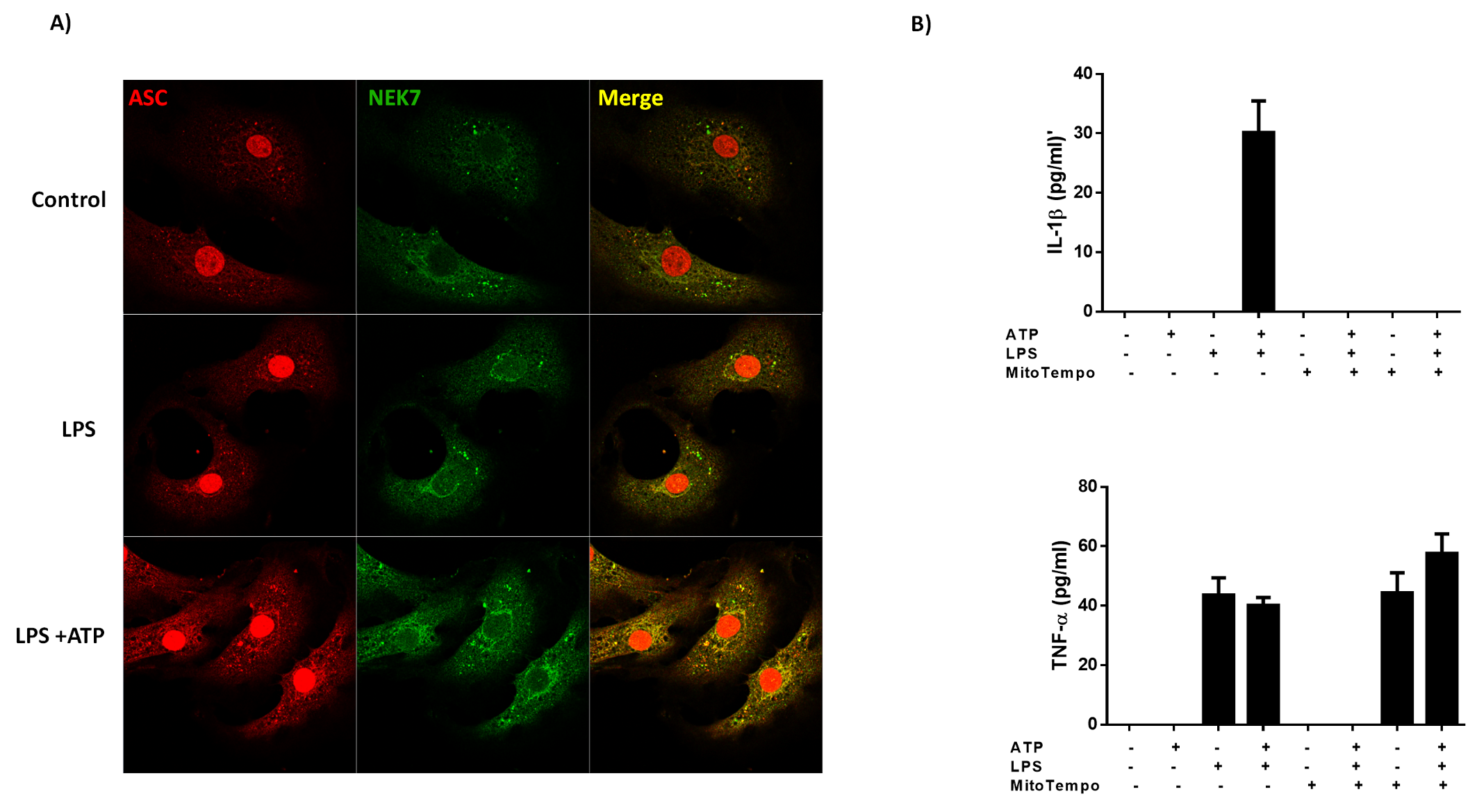

Supplement: Supplementary Figure 1 — A role for NEK7 and mitochondrial ROS in NLRP3-mediated IL-1β release in mouse cardiac fibroblasts. (A) NEK7 co-localizes with ASC after NLRP3-dependent inflammasome formation. Cardiac fibroblasts were primed with 10 ng/mL LPS for 18 h, then activated with 3 mM ATP for 60 min. The cells were fixed and labeled with anti-ASC Alexa 633 and anti-NEK7 Alexa488. (B) The mitochondria specific ROS scavenger MitoTempo inhibits IL-1β release, while TNF-α is not affected. Cardiac fibroblasts were incubated in medium only or 10 ng/ml LPS for 20 h, then mtROS scavenger MitoTempo (100 uM) for 60 min prior to ATP (3 mM) for 60 min. Columns are mean with SEM (n = 3). [file Image_1.TIF]

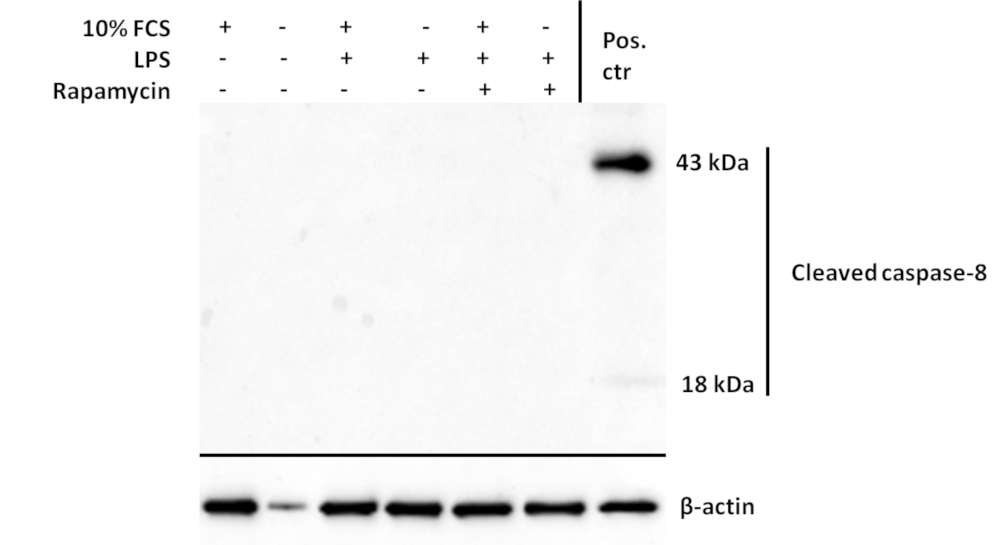

Supplement: Supplementary Figure 2 — No role for caspase-8 in IL-1β release from serum starved cardiac fibroblasts treated with LPS and rapamycin. Cardiac fibroblasts were incubated with or without 10% FCS and primed with 10 ng/mL LPS with or without rapamycin (500 nM) for 20 h prior to activation with ATP for 60 min. Western blot analysis of cleaved caspase-8 were performed. HL-1 cells treated with 10 μM staurosporine for 3 h followed by 22 h incubation in staurosproine-free medium served as positive control. Blot is representative for six independent biological repeats. [file Image_2.TIF]
